# Establishment of a Macrophage Phenotypic Switch Related Prognostic Signature in Patients With Pancreatic Cancer

**DOI:** 10.3389/fonc.2021.619517

**Published:** 2021-03-03

**Authors:** Mu-xing Li, Hang-yan Wang, Chun-hui Yuan, Zhao-lai Ma, Bin Jiang, Lei Li, Li Zhang, Dian-rong Xiu

**Affiliations:** Department of General Surgery, Peking University Third Hospital, Beijing, China

**Keywords:** macrophage phenotype switch, pancreatic ductal adenocarcinoma, prognostic index, TCGA, GEO, GTEx

## Abstract

**Introduction:**

Macrophage phenotype switch plays a vital role in the progression of malignancies. We aimed to build a prognostic signature by exploring the expression pattern of macrophage phenotypic switch related genes (MRGs) in the Cancer Genome Atlas (TCGA)—pancreatic adenocarcinoma (PAAD), Genotype-Tissue Expression (GTEx)-Pancreas, and Gene Expression Omnibus (GEO) databases.

**Methods:**

We identified the differentially expressed genes between the PAAD and normal tissues. We used single factor Cox proportional risk regression analysis, Least Absolute Shrinkage and Selection Operator (LASSO) analysis, and multivariate Cox proportional hazard regression analysis to establish the prognosis risk score by the MRGs. The relationships between the risk score and immune landscape, “key driver” mutations and clinicopathological factors were also analyzed. Gene-set enrichment analysis (GSEA) analysis was also performed.

**Results:**

We detected 198 differentially expressed MRGs. The risk score was constructed based on 9 genes (KIF23, BIN1, LAPTM4A, ERAP2, ATP8B2, FAM118A, RGS16, ELMO1, RAPGEFL1). The median overall survival time of patients in the low-risk group was significantly longer than that of patients in the high-risk group (*P* < 0.001). The prognostic value of the risk score was validated in GSE62452 dataset. The prognostic performance of nomogram based on risk score was superior to that of TNM stage. And GSEA analysis also showed that the risk score was closely related with P53 signaling pathway, pancreatic cancer and T cell receptor signaling pathway. qRT-PCR assay showed that the expressions of the 9 MRGs in PDAC cell lines were higher than those in human pancreatic ductal epithelium cell line.

**Conclusions:**

The nine gene risk score could be used as an independent prognostic index for PAAD patients. Further studies validating the prognostic value of the risk score are warranted.

## Introduction

Pancreatic ductal adenocarcinoma (PDAC), with an estimated 5-year overall survival rate less than 10%, is the fourth leading cause of cancer-related mortality in the world (1). And its incidence is still increasing these years due to lifestyle change and improved medical detection technology. Majority of pancreatic cancer patients were usually at advanced stages at their initial diagnosis. The lack of effective systematic therapies and useful prognostic indexes deteriorates the dismal prognosis of pancreatic cancer patients (2).

With the development of high-throughput technologies, molecular characterization may shed light on newer therapeutic targets (3). Therefore, it is essential to identify molecular prognostic factors of pancreatic cancer which aid in rational stratification of patients according to the clinical prognosis as well as in providing potentially therapeutic targets (4).

It has long been held that the dismal therapeutic effects in pancreatic cancer can largely be attributed to the complex tumor microenvironment (TME). Macrophages are one of the most abundant immune cells in PDAC tumor microenvironment (5). According to their polarization states, macrophages are roughly categorized into two types: classically activated type 1 (M1 macrophages), and alternatively activated type 2 (M2 macrophages) (6). M1 macrophages, characterized by the expression of the inducible-type nitric oxide synthase (iNOS), are pro-inflammatory and develop in response to lipopolysaccharides (LPS) or interferon-γ (IFN-γ). M2 macrophages, or anti-inflammatory macrophages, develop in response to interleukin (IL)-4, IL-13 or glucocorticoids, and are characterized by the secretion of anti-inflammatory mediators, including transforming growth factor-β1 (TGF-β1) and IL-10 to promote extracellular matrix remodeling and angiogenesis (7). M2 macrophages exert pro-tumor functions, whereas M1 macrophages exert anti-tumor functions (8). The macrophage phenotypic switch related gene (MRGs) may provide us with in-depth information on the prognosis of PAAD patients (9).

In the present study, we aimed to build a prognostic model *via* thorough investigation of the cancer genome atlas (TCGA) database, Genotype-Tissue Expression (GTEx) database and Gene Expression Omnibus (GEO) database. We hoped that our prognostic risk score could aid in the prognostic prediction as well as the treatment strategy design.

## Methods

### Data Collection

We collected the transcriptome profiles of pancreatic adenocarcinoma (PAAD) available in the TCGA database (https://portal.gdc.cancer.gov/) and Genotype-Tissue Expression (GTEx) GTEx-Pancreas datasets (https://xenabrowser.net/) on July 5^th^, 2020. Our study included the expression profile of 171 normal samples and 177 PAAD samples. The clinicopathological information including gender, age, tumor grade, T classification, N classification, M classification, TNM stage, follow-up time, and survival status of the patients from TCGA-PAAD was also retrieved (10). We excluded samples with follow-up time shorter than 30 days and samples with missing clinicopathological information. For validation, gene expression data and clinical data of 70 PAAD patients in GSE62452 was downloaded from Gene Expression Omnibus (GEO) database (https://www.ncbi.nlm.nih.gov/geo/). The flow of the study was shown in **Figure S1**.

### Gene Set Selection

Two macrophage phenotype switch related gene sets (GSE5099_CLASSICAL_M1_VS_ALTERNATIVE_M2_MACROPHAGE_UP, GSE5099_CLASSICAL_M1_VS_ALTERNATIVE_M2_MACROPHAGE_DN) (11) were selected from the Molecular Signatures Database v7.1 (MSigDB) (12) (https://www.gsea-msigdb.org/gsea/msigdb/index.jsp). No overlapped genes were detected and a total of 382 genes were retrieved (**Table S1**). The expression data of the 382 MRGs was extracted. Since the data was retrieved from public datasets and we followed the respective publishing guidelines, no ethics approval was needed.

### Screening of Differentially Expressed MRGs

We collected the mean expression data of 382 MRGs comprising 177 PAAD and 171 non-tumor samples. The mean expression values were then normalized by log_2_ transformation. The differentially expressed MRGs between tumor and normal samples were identified using the Wilcoxon signed-rank test in R (version 4.0.2, https://www.r-project.org/) with a threshold of |log(foldchange)| > 1 and a false discovery rate (FDR) < 0.05.

### Identification of the Prognostic Signatures

We built the prognostic signatures in three steps: 1) we conducted univariate Cox regression analysis of the MRGs by “survival” in R. Genes with *P* < 0.05 were chosen for Lasso Cox regression analysis. 2) The Lasso Cox regression analysis (13) which was implemented with “glmnet” and “survival” packages in R was utilized to remove highly correlated genes and to prevent over-fitting. 3) Risk scores were calculated as the sum of each gene’s expression levels multiplying the regression coefficient in the multivariate Cox regression model. Patients were dichotomized into high-risk group and low-risk group by median value of risk score. The Kaplan-Meier method was utilized to compare the survival outcome between high-risk group and low-risk group. Time dependent receiver operating characteristic (ROC) curves by R package “survivalROC” (14) was also used to determine the efficacy of the prognostic model.

We developed a prognostic nomogram predicting OS based on the Cox proportional hazard regression model by the “rms” package in R. A concordance index (C-index) was calculated to evaluate the performance of the nomogram.

### Immune Phenotypes

Using R package “GSVA,” single-sample gene set enrichment analysis (ssGSEA) was conducted to quantify the activity or enrichment levels of immune cells, functions, or pathways (15, 16). The comparison of immune cell distribution between patients at high-risk group and patients at low-risk group was performed.

Besides, the relationships between the MRGs based risk score and several immune checkpoints, including PD-1, PD-L1, CTLA-4, TIM-3, and LAG-3, were analyzed.

### Mutation of the Four Key Drivers

The mutations of the four most prominent key drivers in pancreatic cancer progression, namely KRAS, TP53, CDKN2A, and SMAD4 (17), were downloaded. And the relationships between them and MRGs based risk score were gauged.

### Gene Set Enrichment Analyses

Gene set enrichment analysis (GSEA) (12) was performed in javaGSEA v. 4.1.0 based on the Molecular Signatures Database v. 7.1. C2 (curated gene sets), C5 (GO gene sets), and C6 (oncogenic signatures) were searched to identify enriched KEGG pathways, biological processes, cellular components, molecular functions, and dysregulated oncogenic signatures associated with the high-risk group (12). |NES| > 1 and FDR < 0.05 were considered statistically significant.

### Cell Lines and Culture

Human pancreatic ductal epithelium cell line HPDE6-C7 was obtained from Institute of Biochemistry and Cell Biology, Chinese Academy of Sciences (Shanghai, China). PDAC cell lines (MIA PaCa-2 and Capan-1) were acquired from the American Type Culture Collection (ATCC, Manassas, VA, USA). HPDE6-C7 was cultured in Dulbecco’s modified Eagle’s medium (DMEM) (Invitrogen, Carlsbad, CA, USA) added with 10% fetal bovine serum (FBS) (Invitrogen). MIA PaCa-2 was cultured in DMEM added with 10% FBS and 2.5% horse serum. Capan-1 was cultured in Iscove’s modified Dulbecco’s medium added with 20% FBS. All cells were cultured in a humidified 5% CO_2_ incubator at a temperature of 37°C.

### Quantitative Real-Time Polymerase Chain Reaction

Total RNA was extracted from cells using the TRIzol reagent (Invitrogen). After quantification using NanoDrop 2000c instrument (Thermo Fisher, Waltham, MA, USA), the RNA was reverse transcribed into first-strand cDNA using the M-MLV Reverse Transcriptase (Invitrogen). qRT-PCR was carried out on StepOne Plus Real-Time PCR System (Applied Biosystems, Foster City, CA, USA) using TB Green^®^ Premix Ex Taq™ (Tli RNaseH Plus), ROX plus (TaKaRa, Dalian, China). GAPDH was used as an endogenous control. The quantification of RNA level expression was calculated using the 2^−ΔΔct^ method. The primer sequences used were as follows: for KIF23, 5’-AGTCAGCGAGAGCTAAGACAC-3’ (sense), 5’-GGTTGAGTCTGTAGCCCTCAG-3’ (antisense); for BIN1, 5’-TGAGCAGTGCGTCCAGAATTT-3’ (sense), 5’-CGATCTTGTTTGCCTCATCCC-3’ (antisense); for LAPTM4A, 5’-ATGGTGTCCATGAGTTTCAAGC-3’ (sense), 5’-CCACAGTCAGCAAAATTGCCA-3’ (antisense); for ERAP2, 5’-CACTAATGGGGAACGATTTCCTT-3’ (sense), 5’-CTGACCAAGACTTCGATCTTCTC-3’ (antisense); for ATP8B2, 5’-CGGGCTAATGACCGAGAATAC-3’ (sense), 5’-CTGCTCAAAGAGGTTGACAGG-3’ (antisense); for FAM118A, 5’-GTCGCCCATGATCTGATCCG-3’ (sense), 5’-CTCCAGGTCGTCAAACACCTC-3’ (antisense); for RGS16, 5’-ATCAGAGCTGGGCTGCGATA-3’ (sense), 5’-CAGGTCGAACGACTCTCTCC-3’ (antisense); for ELMO1, 5’-GGAGCAGGTTATGAGAGCACT-3’ (sense), 5’-GGGCGGGACTGGAAATCTTC-3’ (antisense); for RAPGEFL1, 5’-AGGGGCTGCTTCAAGAGGA-3’ (sense), 5’-CCCTGGTAAAGGGACTCGT-3’ (antisense).

### Statistical Analysis

All statistical analyses were conducted using R software (version 4.0.2), GraphPad Prism 7 (San Diego, CA, USA), and SPSS 20.0 (Chicago, IL, USA). The median OS was compared using the Kaplan-Meier method with log-rank test. The distributions of clinicopathological parameters between the high-risk and low-risk groups were compared using chi-square tests, T test, or one-way analysis. Two-sided *P* value less than 0.05 was considered as statistically significant.

## Results

### Identification of Differentially Expressed MRGs

**Figure 1:**
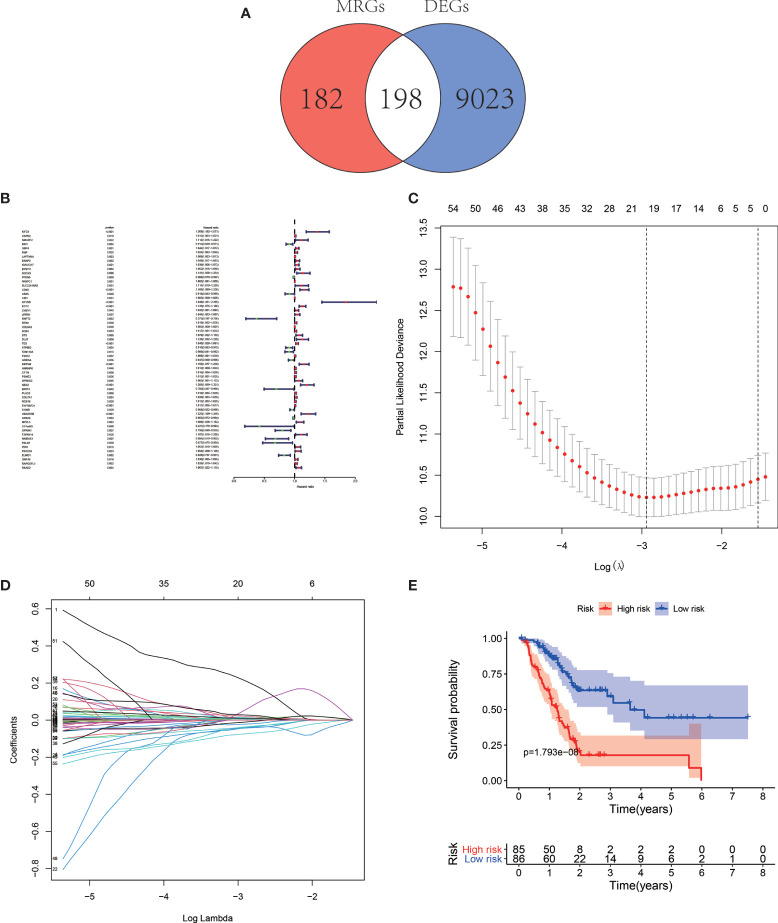
Venn map illustrating the identification of the differentially expressed MRGs **(A)**. Forest map of MRGs related to pancreatic adenocarcinoma (PAAD) survival by univariate Cox regression analysis **(B)**. Least Absolute Shrinkage and Selection Operator (LASSO) coefficient spectrum of the nine MRGs selected by the univariate Cox regression. Generate a coefficient distribution map for a logarithmic (λ) sequence **(C)**. Selecting the best parameters for PAAD in the LASSO model (λ) **(D)**. Kaplan–Meier survival analysis of TCGA-PAAD patients stratified by the median value of risk score **(E)**.

In total, 198 differentially expressed MRGs meeting the criteria as |log(foldchange)| > 1 and FDR < 0.05 by the pooled analysis of TCGA-PAAD and GTEx-pancreas databases were obtained (**Figure 1A**, **Table S2**).

### Survival−Related MRGs and the Prognostic Signature

The univariate Cox regression analysis and identified 59 MRGs that were significantly related to overall survival in TCGA-PAAD cohort (**Figure 1B**). The Lasso regression analysis excluded 40 genes that may be highly correlated with other genes (**Figures 1C, D**). The rest 19 genes (KIF23, BIN1, GBP4, LAPTM4A, ERAP2, SLC22A18AS, CDK6, KIF20B, RNFT2, TES, ATP8B2, FAM118A, ARID5A, RGS16, TNFRSF21, UBASH3B, GPRIN1, ELMO1, RAPGEFL1) were then submitted to a multivariate Cox proportional hazards model, resulting in 9 candidate genes (KIF23, BIN1, LAPTM4A, ERAP2, ATP8B2, FAM118A, RGS16, ELMO1, RAPGEFL1) as significant predictors of prognosis (**Table 1**). The formula for the risk score of every PAAD patient was constructed: risk score = (expression value of KIF23 * 0.394) − (expression value of BIN1 * 0.098) + (expression value of LAPTM4A * 0.005) + (expression value of ERAP2 * 0.051) − (expression value of ATP8B2 * 0.082) − (expression value of FAM118A * 0.081) + (expression value of RGS16 * 0.014) − (expression value of ELOM1 * 0.112) + (expression value of RAPGEFL1 * 0.017).

**Table 1:** The 9 MRGs in the prognostic risk score by the LASSO multivariate Cox proportional hazards model.

Gene	Full name	Coefficient	HR	*P*
KIF23	kinesin family member 23	0.393637	1.482362	0.000629
BIN1	bridging integrator 1	-0.09805	0.906601	0.013274
LAPTM4A	lysosomal protein transmembrane 4 alpha	0.00464	1.004651	0.12958
ERAP2	endoplasmic reticulum aminopeptidase 2	0.050903	1.052221	0.010708
ATP8B2	ATPase phospholipid transporting 8B2	-0.08246	0.920848	0.080459
FAM118A	family with sequence similarity 118 member A	-0.08058	0.922586	0.037329
RGS16	regulator of G protein signaling 16	0.014164	1.014264	0.023283
ELMO1	engulfment and cell motility 1	-0.11243	0.893659	0.028348
RAPGEFL1	Rap guanine nucleotide exchange factor like 1	0.017138	1.017286	0.082629

HR, Hazard ratio.

**Figure 2:**
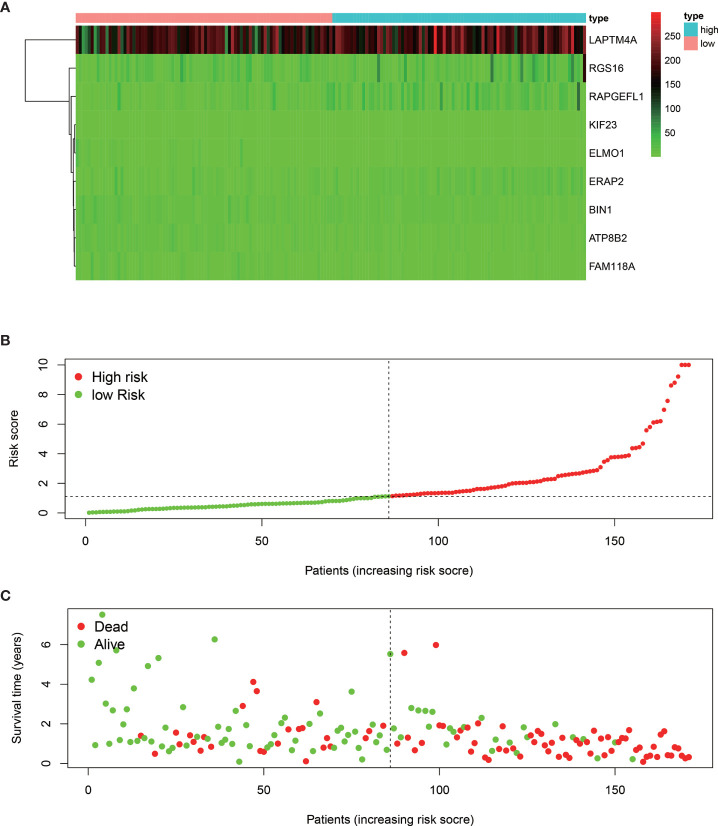
Heat map of the nine MRGs expression profiles stratified by the risk score **(A)**. Distribution of the patients along with the increasing of risk score **(B)**. Distribution of patients’ survival status **(C)** stratified by risk score. The black dotted line is the cut-off value (median value) for dividing patients into low-risk and high-risk groups.

**Figure 3:**
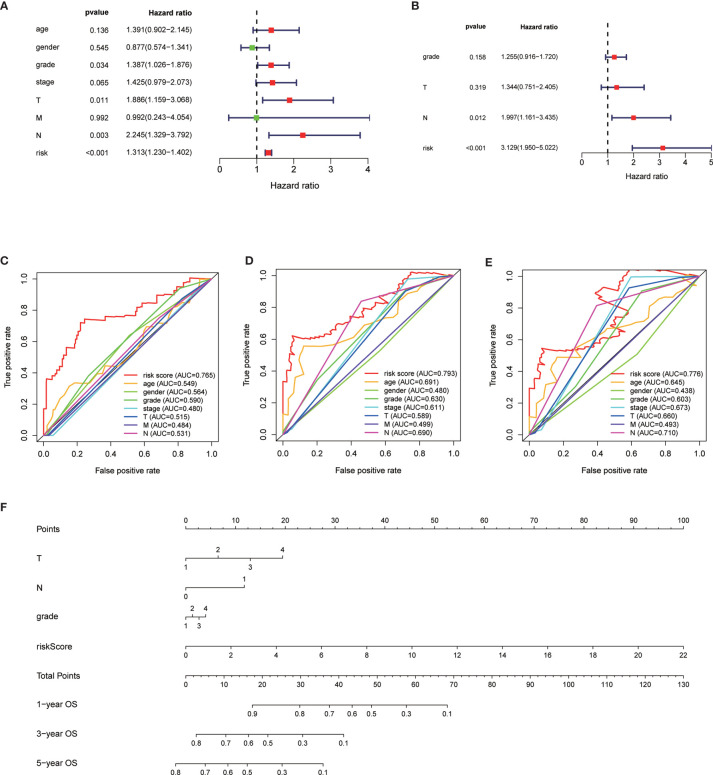
Univariate analysis of risk score and other clinicopathological factors predicting the overall survival **(A)**. Multivariate analysis of risk score and other clinicopathological factors predicting the overall survival **(B)**. Time dependent receiver operating characteristic (ROC) curve of risk score and other clinicopathological factors predicting the 1-year overall survival **(C)**, 3-year overall survival **(D)**, and 5-year overall survival **(E)** in The Cancer Genome Atlas (TCGA)-pancreatic adenocarcinoma (PAAD) cohort. The prognostic nomogram based on the risk score predicting the OS **(F)**.

The median overall survival time of patients in the high-risk group was significantly lower than that in the low-risk group (**Figure 1E**, *P* < 0.001). Expression profile of the included 9 MRGs (**Figure 2A**), distribution of patients (**Figure 2B**), individual survival status (**Figure 2C**) stratified by risk score were shown in **Figure 2**. Risk score remained to be an independent prognostic indicator in multivariate analysis (HR=3.129, 95% CI = 1.950–5.022, *P* < 0.001, **Table 2**, **Figures 3A, B**). Then we assessed the prediction efficiency of the risk score by drawing the time dependent ROC curve, which revealed that the risk score could predict the 1-year overall survival (AUC=0.765, **Figure 3C**), 3-year overall survival (AUC =0.793, **Figure 3D**), and 5-year overall survival (AUC =0.776, **Figure 3E**) for PAAD patients effectively. And the AUC of risk score was larger than those of the other enrolled clinocopathological factors.

**Table 2:** Univariate and multivariate analysis of the overall survival in The Cancer Genome Atlas-pancreatic adenocarcinoma (TCGA-PAAD) patients.

Variable	Number	Univariate analysis	Multivariate analysis		
		*P*	HR	95% CI	*P*
Age (years)		0.136			
≥ 60	114				
< 60	52				
Gender		0.545			
Male	90				
Female	76				
Tumor grade		**0.034**	1.255	0.916–1.720	0.158
1	26				
2	91				
3	47				
4	2				
T classification		**0.011**	1.344	0.751–2.405	0.319
1	6				
2	21				
3	136				
4	3				
N		**0.003**	**1.997**	**1.161–3.435**	**0.012**
0	49				
1	117				
M classification		0.992			
0	162				
1	4				
TNM stage		0.065			
I	18				
II	141				
III	3				
IV	4				
Riskscore		**<0.001**	**3.129**	**1.950–5.022**	**<0.001**
High	83				
Low	83				

Significant results were expressed in bold.

### Validation of the Risk Signature

**Figure 4:**
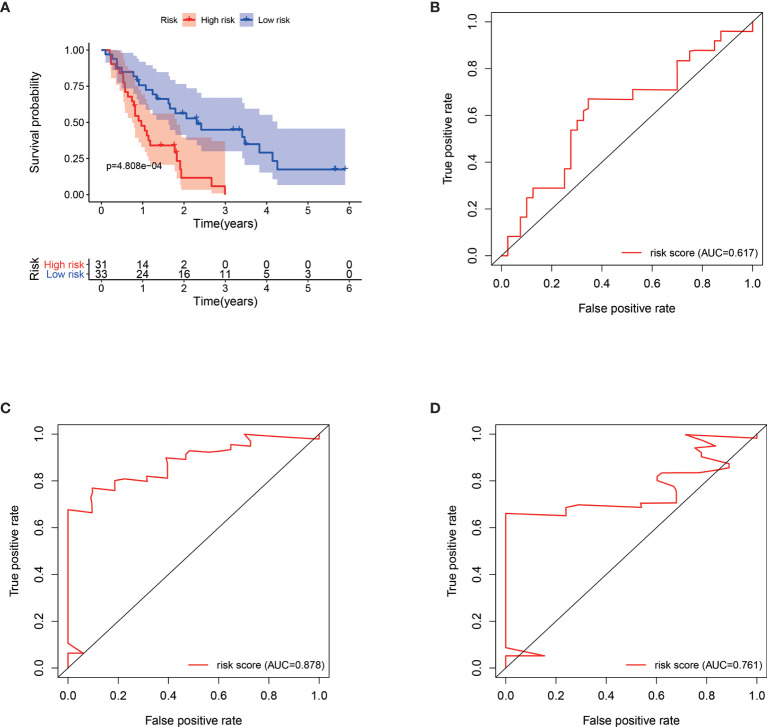
Kaplan–Meier survival analysis of GSE62452 patients stratified by the median value of risk score **(A)**. Time dependent receiver operating characteristic (ROC) curve of risk score predicting the 1-year overall survival **(B)**, 3-year overall survival **(C)**, and 5-year overall survival **(D)** in GSE62452 patients.

We verified the robustness of our MRG based risk score with the patients from GSE62452 dataset. We calculated the risk score for patients and divided them into high-risk group and low-risk group in test dataset with the same formula derived from the training cohort as the TCGA-PAAD database. Patients in low-risk group had obviously better overall survival (*P* < 0.001, **Figure 4A**). After adjusting for clinicopathological features, risk score remained to be an independent prognostic indicator in multivariate analysis (HR=2.385, 95% CI = 1.209-4.707, *P* = 0.012, **Table 3**). Then we assessed the prediction efficiency of the risk signature by drawing the time dependent ROC curve, which revealed that the risk score could predict the 1-year overall survival rate (AUC = 0.617, **Figure 4B**), 3-year overall survival rate (AUC = 0.878, **Figure 4C**), and 5-year overall survival rate (AUC = 0.761, **Figure 4D**) for GSE62452 dataset effectively.

**Table 3:** Univariate and multivariate analysis of the overall survival in GSE62452 patients.

Variable	Number	Univariate analysis	Multivariate analysis		
		*P*	HR	95% CI	*P*
Tumor grade*		**0.019**	1.470	0.890–2.429	0.133
1	2				
2	31				
3	29				
4	1				
TNM stage		0.560			
I	4				
II	44				
III	10				
V	6				
Riskscore		**<0.001**	**2.385**	**1.209–4.707**	**0.012**
High	33				
Low	31				

*The information of tumor grade was unknown in one patient. Significant results were expressed in bold.

### Construction of the Nomogram

The 166 patients with complete clinical information from the TCGA-PAAD dataset were used to establish a prognostic nomogram based on the stepwise Cox regression model (**Figure 3F**). The MRGs based risk score, T classification, and N classification were finally included into the stepwise Cox regression model for the nomogram. The C-index of the nomogram was 0.723, which was higher than that of TNM stage itself as 0.535. Thus, the nomogram was regarded to be superior to the TNM stage in terms of predicting overall survival of PAAD.

### Relationship Between MRGs Risk Score and Immune Landscape

**Figure 5:**
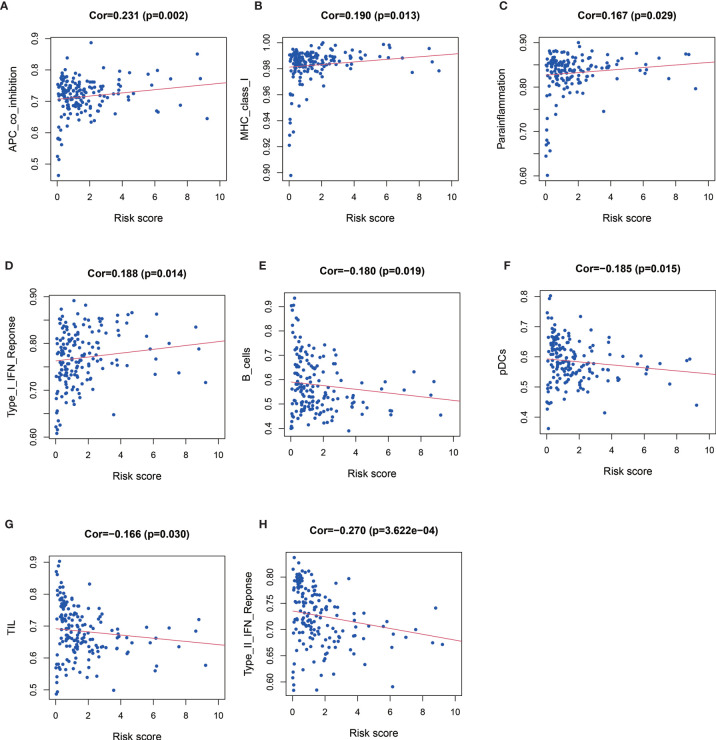
Relationships between risk score and APC co-inhibition **(A)**, major histocompatibility complex (MHC) class I **(B)**, parainflammation **(C)** and type I IFN response **(D)**, B cell **(E)**, pDCs **(F)**, TIL **(**Figure **G)**, and type II IFN response **(H)**.

Patients with high risk score had significantly higher level of APC co-inhibition (Cor=0.231, *P* = 0.002, **Figure 5A**), MHC class I (Cor=0.190, *P*=0.013, **Figure 5B**), parainflammation (Cor=0.167, *P*=0.029, **Figure 5C**), and type I IFN response (Cor=0.188, *P*=0.014, **Figure 5D**), but significantly lower level of B cell (Cor = −0.180, *P* = 0.019, **Figure 5E**), pDCs (Cor = −0.185, *P* = 0.015, **Figure 5F**), TIL (Cor = −0.166, *P* = 0.030, **Figure 5G**), and type II IFN response (Cor = −0.270, *P* < 0.001, **Figure 5H**).

**Figure 6:**
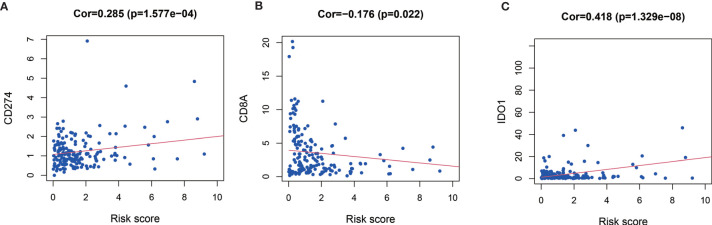
Relationships between risk score and CD274 (PD-L1) **(A)**, CD8A **(B)**, and IDO1 **(C)**.

Relationship between expression level of co-inhibitory immune checkpoints (PD-1, PD-L1, CTLA-4, TIM-3, LAG-3, CD8A) and MRGs based risk score were also assessed. Patients with high risk score had significantly higher level of CD274 (Cor=0.285, *P* < 0.001, **Figure 6A**), IDO1 (Cor=0.418, *P* < 0.001, **Figure 6C**), but significantly lower level of CD8A (Cor = −0.176, *P* = 0.022, **Figure 6B**).

### Relationship Between MRGs Risk Score and “Key Driver” Mutations

**Figure 7:**
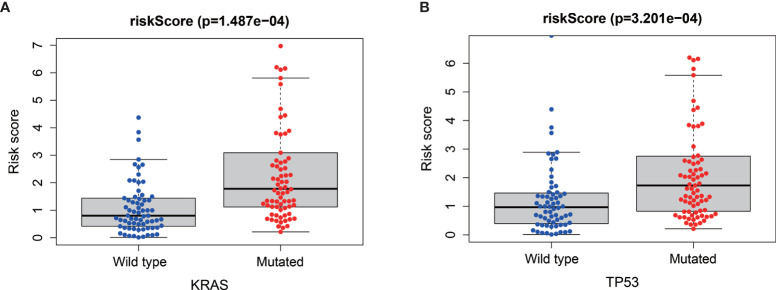
Relationships between risk score and KRAS mutation **(A)** and TP53 mutation **(B)**.

*Via* the analysis of the relationship between the mutations of the four “key drivers” of pancreatic cancer and risk score, we found that patients with higher risk score were enriched for KRAS mutations (*P*<0.001, **Figure 7A**) and TP53 mutations (*P* < 0.001, **Figure 7B**).

### Relationship Between MRGs Risk Score and Clinicopathological Features

**Figure 8:**
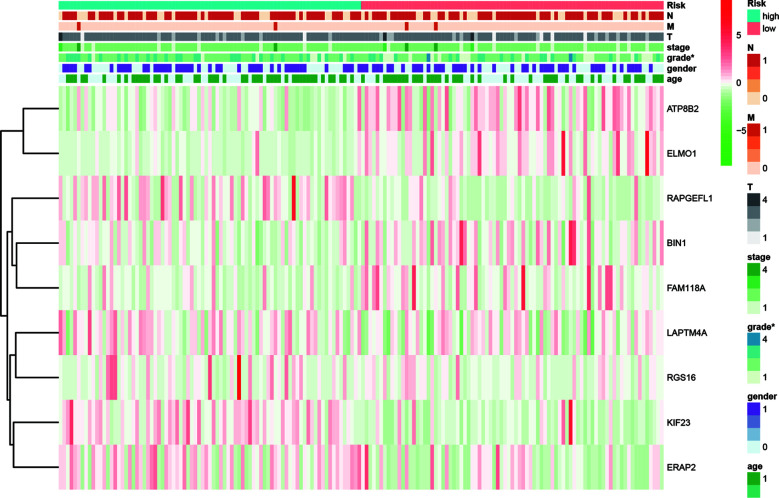
Relationships between risk score and the enrolled MRGs expression, survival outcome, and clinicopathological factors.

Relationship between MRGs prognostic index and clinicopathological features were subsequently analyzed (**Figure 8**). Significant higher risk scores were in patients with higher tumor grade (*P* < 0.001). In addition, the expression level of BIN1 was significantly related with advanced M (*P* < 0.001). The expression level of ELMO1 was significantly related with higher tumor grade (*P* = 0.008). The expression level of ERAP2 was significantly related with advanced N (*P* = 0.027). The expression level of KIF23 was significantly related with higher tumor grade (*P* < 0.001). The expression level of LPTM4A was significantly related with younger age (*P*=0.038) and higher tumor grade (*P*=0.024). The expression level of RGS16 was significantly related with higher tumor grade (*P*=0.015).

### Gene Set Enrichment Analysis

**Figure 9:**
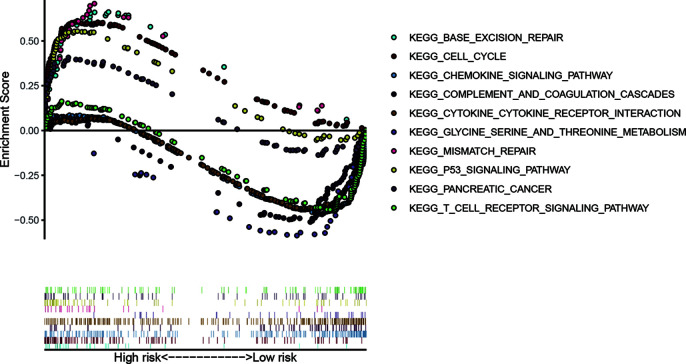
Gene-set enrichment analysis (GSEA) compared the high- and low risk groups stratified by the median value of risk score.

GSEA compared the high- and low risk groups stratified by the risk score (**Figure 9**). KEGG pathways enriched in the high-risk group included KEGG P53 signaling pathway, KEGG cell cycle, KEGG base excision repair, KEGG pancreatic cancer, and KEGG mismatch repair. KEGG pathways enriched in the low-risk group included KEGG glycine serine and threonine metabolism, KEGG chemokine signaling pathway, KEGG complement and coagulation cascades, KEGG cytokine cytokine receptor interaction, and KEGG T cell receptor signaling pathway.

### Expressions of the Nine MRGs in PDAC Cell Lines

**Figure 10:**
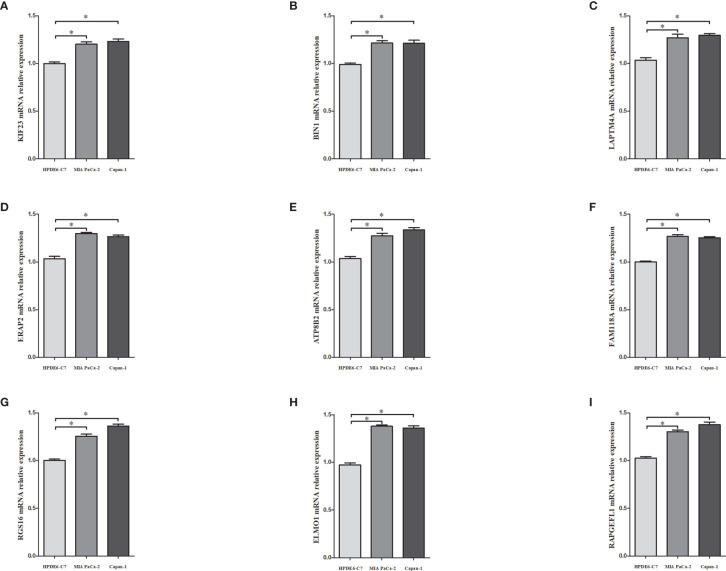
The mRNA expressions of KIF23 **(A)**, BIN1 **(B)**, LAPTM4A **(C)**, ERAP2 **(D)**, ATP8B2 **(E)**, FAM118A **(F)**, RGS16 **(G)**, ELMO1 **(H)**, RAPGEFL1 **(I)** in pancreatic ductal epithelium cell line (HPDE6-C7) and PDAC cell lines (MIA PaCa-2 and capan-1). * denoted that P<0.05.

The mRNA expressions of the nine MRGs (KIF23, BIN1, LAPTM4A, ERAP2, ATP8B2, FAM118A, RGS16, ELMO1, RAPGEFL1) in pancreatic ductal epithelium cell line (HPDE6-C7) and PDAC cell lines (MIA PaCa-2 and Capan-1) were assessed. Transcriptional expressions of the 9 MRGs were significantly higher in PDAC cell lines than those in HPDE6-C7 (**Figure 10**).

## Discussion

The present study, to the best of our knowledge, was the first study constructing a prognostic index based on MRGs *via* analyzing the TCGA, GTEx, and GEO datasets. We identified nine prognostic MRGs. We evaluated the prognostic value of the nine-gene signature and constructed a nomogram. Our results suggested the promising value of tumor-associated macrophage in pancreatic cancer.

Due to its simplicity and practicality, TNM staging system remains to be the most widely used cancer staging system. It acts as the base for the prognosis prediction and the selection of therapeutic options (18). Meanwhile, diverse clinical outcomes may be observed in patients at the same TNM stage. Along with the development of precision medicine, it is increasingly recognized that the molecular biomarkers provide important prognostic information and clinical implication. With the development of large-scale gene sequence databases, some molecular prognostic features have been proposed. In the 8^th^ AJCC staging manual for breast cancer (19), the gene panel have been introduced into the staging system, which partly reflects the increasing recognization of the value of molecular prognostic factors.

Immune dysregulation is pivotal in cancer progression. To date, researches on the immune dysregulation have largely focused on the T cell compartment (20, 21). Other immune subsets including macrophages, which constitute the largest portion of immune cells in TME, may also contribute to the cancer progression (22). In our study, we discovered that high risk score was associated with increased parainflammation and APC co inhibition. Parainflammation, which was initially described by Pribluda et al., referred to the moderately increased inflammatory cytokines which might promote the proliferation, angiogenesis, invasion, and migration. Parainflammation is thus considered as a promotion factor in carcinogenesis (23, 24). Further, increased APC co-inhibition, along with the increase of MRGs based risk score, partly reflects the defected neoantigen recognition, presentation, and anti tumor effects. Meanwhile, positive relationship between risk score and MHC class I indicates increased recognition by cytotoxic T cells (25). The effects may be compensated by the complicated immune network involving parainflammation and APC co-inhibition. The negative relationship between MRGs risk signature and B cells also suggests defected immune response. Of note, the positive relationship between risk score and type I interferon (IFN) response while the negative relationship between risk score and type II IFN response are intriguing. We speculated that the complicated biological function of the two types of IFN responses might accelerate the tumor development (26, 27) in patients with higher MRGs based risk score, which called for more researches in the future.

Immune checkpoints play a crucial role in carcinogenesis by promoting tumor immunosuppressive effects (28). Tumors can protect themselves from being attacked by stimulating immune checkpoint targets. In our study, immune checkpoints expressing in the tumor tissues as PD-L1 were upregulated in the group with higher MRGs based risk score. PD-L1, also known as CD274, is one of the ligands that binds to programmed death-1 (PD-1) on T cells and attenuates the immune response by downregulating the activity of antitumor T cells. In the PDAC microenvironment, PD-L1 is highly expressed in cancer cells, facilitating immune escape and cancer progression (29). Blockade of PD-L1 has gained survival benefits in various cancers including the NSCLC, myeloma, and kidney cancer. However, a previous study indicated that targeting PD-L1 for PDAC therapy was unsuccessful, as the response rate was < 3.1% (28, 29). It was suggested that the macrophage phenotypic switch might compensate the effects by blocking of PD-L1 (30, 31). The positive relationship between MRGs risk score and IDO1 also referred to defected anti-tumor immune response (32). The decreased CD8A expression indicated defected CD8 T cell infiltration and cytolytic effects (33). Our results further revealed the potential influence of macrophage phenotypic switch on immune checkpoints and immune escape.

There are four major driver genes for pancreatic cancer, including KRAS, CDKN2A, TP53, and SMAD4 (17). KRAS mutations occurred in the early stage of pancreatic cancer. As a classic tumor suppressor gene, TP53 has mutation in many tumor types. In our study, we found that the mutation rates of KRAS and TP53 in the high-risk group was significantly higher than that in the low-risk group, which suggested that macrophage phenotype switch might be related to somatic mutations (34). Besides, Bishehsari et al. reported that KRAS mutations induced a protumorigenic phenotype in macrophages (35). These findings were consistent with our results.

The strength of our study came as it shifted from the T cell compartment to the macrophage compartment. We explored the relationship between the 9 MRGs risk score and immune landscape, “key driver” mutations and so on. And some basic experiments were performed. Admittedly, there were some limitations in this study. As a retrospective study, though we validated the nine MRGs risk score using training/testing sets from the public databases, it was limited by the selection bias. Secondly, the external validation of our risk signature in GEO datasets strengthened the robustness of our results. We should remind that more external validation was needed. Thirdly, we tested the expression of the MRGs in the cell line experiments. The biological processes and molecular mechanisms of the nine MRGs should be in depth evaluated in more *in-vivo* and *in-vitro* studies.

## Conclusions

Our study suggested that nine macrophage phenotypic switch related gene signature had satisfactory prognostic ability. The nine-gene signature may provide us with a novel metric for prognosis prediction and more potential treatment targets for pancreatic cancer.

## Data Availability Statement

The datasets presented in this study can be found in online repositories. The names of the repository/repositories and accession number(s) can be found in the article/**Supplementary Material**.

## Author Contributions

M-xL and H-yW conceived and performed the study. C-hY, Z-lM, BJ, and LL guided the analysis. LZ polished the manuscript. D-rX guided the research process. All authors contributed to the article and approved the submitted version.

## Funding

This study was supported by Key Clinical Projects of Peking University Third Hospital (No. BYSY2018025) and by the Capital Characteristic Clinical Application Research and Achievement Promotion Project (No. Z171100001017121).

## Conflict of Interest

The authors declare that the research was conducted in the absence of any commercial or financial relationships that could be construed as a potential conflict of interest.

## Supplementary Material

Supplementary Table 1 The MRGs from Molecular Signatures Database v7.1 (MSigDB).

Supplementary Table 2 The differentially expressed MRGs by the pooled analysis of TCGA-PAAD and GTEx-pancreas.

Supplementary Figure 1 The flowchart of the study.

## Abbreviations

MRGs, macrophage phenotypic switch related genes; TCGA, The Cancer Genome Atlas; PAAD, pancreatic adenocarcinoma; GTEx, Genotype-Tissue Expression; GEO, Gene Expression Omnibus database; KEGG, Kyoto encyclopedia of Genes and Genomes; GSEA, gene-set enrichment analysis; TNM, tumor-node-metastasis; AJCC, American Joint Committee on Cancer; qRT-PCR, quantitative real-time polymerase chain reaction.
