# Supplementary material for: Establishment of a Macrophage Phenotypic Switch Related Prognostic Signature in Patients With Pancreatic Cancer
Source: Front Oncol. 2021 Mar 3;11:619517. doi: 10.3389/fonc.2021.619517 (PMC7966706; doi:10.3389/fonc.2021.619517)
Supplement: Supplementary Table 2 — The differentially expressed MRGs by the pooled analysis of TCGA-PAAD and GTEx-pancreas. [file DataSheet_2.doc]

Table S2 The differentially expressed MRGs by the pooled analysis of TCGA-PAAD and GTEx-pancreas.

| Gene name | Gene name | Gene name | Gene name |
| --- | --- | --- | --- |
| SORT1 | GNAQ | GXYLT2 | PLAC8 |
| KIF23 | NFKBIA | LIPA | COL7A1 |
| WFDC3 | CEPT1 | DBN1 | HLA-G |
| MSI2 | TMEM128 | FCGR2C | RGS16 |
| RNF213 | CIB1 | CD1D | RPLP1 |
| EIF4E2 | CTSL | IL12RB1 | TNFRSF21 |
| GALNT7 | LMO2 | COX7C | CRNDE |
| CAPN2 | KIF20B | ATP8B2 | ADGRB2 |
| ITIH3 | COL1A2 | BMS1 | S100B |
| CD79B | TRIM69 | FAM118A | ATP10A |
| GGT1 | LRRC8D | HOXA10 | IL27RA |
| SMURF2 | ECT2 | MARCKSL1 | GPA33 |
| BIN1 | BCL11B | P4HA1 | RASA3 |
| GIMAP2 | CHSY1 | ARID5A | UBASH3B |
| GBP4 | ANP32A | UBALD2 | ANXA6 |
| HCP5 | OSBPL5 | IMPACT | CD27 |
| TXLNGY | EPB41L2 | RPS6KA3 | LDLRAD3 |
| PNP | ZNFX1 | CCR7 | TUBA4A |
| LAPTM4A | HMGB3 | RPLP2 | MPZL3 |
| CHD9 | TCF7 | USP9Y | DST |
| AP3M2 | LITAF | ALDH2 | UPP1 |
| ERAP2 | MLLT3 | S100A6 | YWHAQ |
| KIAA1217 | LRRN1 | IL4R | ADD3 |
| FUCA1 | CERS2 | POMP | SERTAD4-AS1 |
| CORIN | GPR4 | INPP4B | C17orf49 |
| EPSTI1 | AKR1C3 | HNRNPK | SPON1 |
| LYST | TLK1 | CNOT11 | ANOS1 |
| SUSD3 | TOMM5 | TPP2 | EEF1G |
| BTG1 | TSPAN11 | CTTN | SPCS2 |
| CFP | S1PR1 | PSME2 | GPRIN1 |
| CD300A | RNFT2 | CENPV | TSPAN14 |
| DOCK9 | RTN4 | SEMA4C | PCGF5 |
| GIMAP8 | BTN3A3 | GYPC | NKIRAS1 |
| TRMT10C | APRT | FLNA | LILRA5 |
| PFDN5 | COL6A3 | PALLD | RGS1 |
| APOL3 | LAMP3 | CD55 | CD48 |
| PCMT1 | SOX4 | SIGLEC9 | FBLN7 |
| PABPC1 | SMPD3 | EIF3K | IFIH1 |
| MFNG | STS | CBLB | RPL30 |
| SLC22A18AS | CMC2 | ZFAND6 | PDE4B |
| FOXO1 | DLAT | HNRNPA2B1 | FBXO34 |
| ZBTB12 | KDM5D | TFPI | ELMO1 |
| COTL1 | TES | LRRC8C | GNA15 |
| CDK6 | TFF3 | SPRED2 | RAPGEFL1 |
| RASSF2 | AEBP1 | CNIH4 | RSAD2 |
| CBX5 | LPIN2 | GJC1 | MAF |
| SEMA3A | CTSZ | FZD1 | HLA-B |
| FOXP1 | DNAJC9 | NEK2 | LPIN1 |
| PLEKHO1 | RGS19 | BAG3 |  |
| PTGS1 | PLEKHA2 | BATF3 |  |
