# Supplementary figures and images for: Establishment of a Macrophage Phenotypic Switch Related Prognostic Signature in Patients With Pancreatic Cancer
Source: Front Oncol. 2021 Mar 3;11:619517. doi: 10.3389/fonc.2021.619517 (PMC7966706; doi:10.3389/fonc.2021.619517)

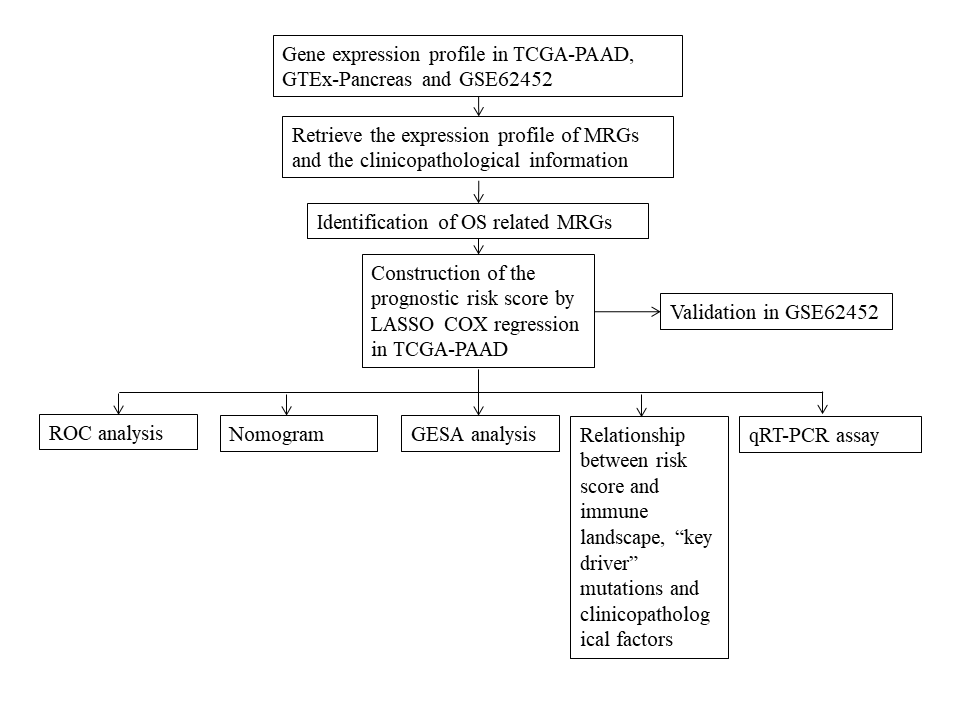

Supplement: Supplementary Figure 1 — The flowchart of the study. [file Image_1.tif]
